# Physical Activity Levels of Adult Virtual Football Players

**DOI:** 10.3389/fpsyg.2021.596434

**Published:** 2021-03-26

**Authors:** Ana M. Pereira, Evert Verhagen, Pedro Figueiredo, André Seabra, António Martins, João Brito

**Affiliations:** ^1^Portugal Football School, Portuguese Football Federation, FPF, Cruz Quebrada, Portugal; ^2^Research Center in Sports Sciences, Health Sciences and Human Development (CIDESD), University Institute of Maia, ISMAI, Maia, Portugal; ^3^Amsterdam Collaboration for Health and Safety in Sports, Department of Public and Occupational Health, Amsterdam Movement Sciences, Amsterdam (UMC), University Medical Centers – Vrije Universiteit Amsterdam, Amsterdam, Netherlands; ^4^Research Centre in Physical Activity, Health and Leisure, Faculty of Sport, University of Porto, Porto, Portugal; ^5^Public Health Unit of Alto Ave, North Regional Health Administration, Porto, Portugal

**Keywords:** esports, video games, physical activity, exercise and health, virtual football

## Abstract

Esports, including virtual football, are a worldwide phenomenon. Yet, little is known about the physical activity levels of individuals engaged in virtual football game play. Therefore, we aimed to perform a preliminary evaluation of the levels of physical activity, sedentarism, and habits of physical training of adults engaged with virtual football in Portugal. This was a cross-sectional investigation based on a structured online survey using the International Physical Activity Questionnaire (IPAQ) and a set of questions regarding habits of physical training. The participants (*n* = 433) reported spending a median of 5,625 MET-min⋅week^−1^ being physically active. Still, the participants spent 320 min/day sitting, and 150 min/day practicing virtual football. According to the IPAQ scores, high physical activity levels were reported by 84.5% of the participants, and 87.1% were considered physically active considering the WHO guidelines on physical activity and sedentary behavior. Overall, 60.0% of the participants reported planning their own physical training. Maintaining or improving overall physical health was one of the main reasons for doing physical training (66.7%), with only 6.1% responding being active to improve virtual football performance. Overall, the results showed that virtual football players accomplished the standard recommendations for physical activity, with high levels of physical activity, and encompassing regular physical training focused mostly on health promotion, rather than improved virtual football performance.

## Introduction

Esports, or electronic sports, comprises a group of different videogame genres (e.g., *sports, first-person shooter, etc.*) played on a specific environment (i.e., in-site, on-line or both), mostly in national and international competitive tournaments (Karhulahti, 2017; Hallmann and Giel, 2018; Geoghegan and Wormald, 2019; Pereira et al., 2019). Generally, esports are organized video game competitions set by rules that require skill and have a broad following; however, esports currently lack great physicality and institutionalization (Jenny et al., 2016). Over the last few years, the interest in esports has increased. Not only by players, but also esports enthusiasts, spectators and investors (Cunningham et al., 2018). Moreover, different scientific fields (e.g., marketing, law, exercise and health) also started to elaborate on the topic, turning esports into a fruitful topic for scientific research. In 2017, there were more than 215 million frequent esports viewers and enthusiasts (WePC, 2019). Even though only a few esports players can reach the professional level (Nielsen and Karhulahti, 2017), in many countries, for some esports players their practice goes beyond just playing videogames as a hobby (DiFrancisco-Donoghue et al., 2019). Portuguese esports players are entering the realm of esports and their level grows; thus, it is important to know more about screen time, inactivity and sedentary behavior due to esports, with no distinction between recreative or professional esports players. Excessive screen time, inactivity and sedentarism behavior are a global public health problem and have been associated with negative short- and long-term health-related problems, such as cardiovascular diseases, mental problems and cancer (Guthold et al., 2008; Hallal et al., 2012; U.S. Department of Health and Human Services, 2018). Contrarily, high levels of physical activity have been associated with a reduction in mortality and major comorbidities (Melzer et al., 2004; Lear et al., 2017; Piercy et al., 2018; World Health Organization, 2020).

Interestingly, recent studies on physical activity levels of videogame and esports players stated that esports game play and involvement-related physical activity is high and could be a driving force to enhanced physical activity for both players and viewers (Pereira et al., 2019; de Las Heras et al., 2020). This would be especially relevant in those esports related to traditional sports (Ballard et al., 2009), such as virtual football. Actually, esports players at elite levels of different esports genres have been reported to be physically active, which is against the empirical notion that esports players are inactive (Kari and Karhulahti, 2016; Trotter et al., 2020). Besides, exercise and physical activity might be important to increased esports performance (de Las Heras et al., 2020; Toth et al., 2020). With Portuguese esports scene rising (Fepodele, 2019)—with special emphasis in virtual football –, it is vital to know more about the physical activity levels of people engaged with esports and understand the relationship between esports practice, sedentary time and motivations for being physically active. Therefore, the aim of the current preliminary study was to characterize and describe the physical activity levels and physical training habits of people engaged in virtual football, a popular esports genre related to a traditional sport (i.e., soccer).

## Materials and Methods

### Participants and Procedures

The present preliminary study was conducted to fulfill a preliminary approach to understanding how to start scientific research in virtual football players in Portugal, and how to improve the methodologies and research designs for the forthcoming investigations. It involved a convenience sample of virtual football players registered on the Portuguese Football Federation (FPF) Esports on-line platform in May 2018. The study employed a cross-sectional design based on a structured online survey comprising of two parts. Part 1 included the Portuguese version of the International Physical Activity Questionnaire—short-form (IPAQ-SF) (IPAQ group, 2005); and Part II consisted of a set of questions regarding habits and motivations for physical training. To be included in the study, the participants had to fulfill the following inclusion criteria: being registered on the FPF Esports platform and aged ≥18 and <65 years [according to the International Physical Activity Questionnaire—short-form (IPAQ-SF) (IPAQ group, 2005) and to the recommendations for physical activity proposed by the WHO for adults (World Health Organization, 2020)]. Also, the complete response to every relevant IPAQ question and responses to all applicable questions related to habits of physical training were the criteria for entering the analysis for each part of the questionnaire. To be registered in the FPF Esports platform, the participants were only required to give the name, birth date and e-mail account, disregarding being a regular or one-time player. The registration did not categorize the participants by occupation or level of play, with no added value separating professional and recreational players. Instead, daily practice time was assumed as esports commitment. All participants were previously informed about the objectives of the study and a written informed, voluntary, consent was provided in the invitation to participate. Participation in the study was entirely voluntary and privacy rights were protected. The study was approved by the Data Protection Officer of the FPF and the Ethics Committee of the Faculty of Sports, University of Porto (CEFADE/31/2019).

A link to the online survey was sent via e-mail to all the 5,748 virtual football players registered with FPF Esports platform (99% men, *n* = 5,687; and 1% female, *n* = 61). The questionnaire was available via SurveyMonkey^®^ for a period of 1 week. The estimated time to complete the questionnaire was previously estimated to be 2–4 min. Overall, 926 participants (16% of the invited sample) accepted to participate and met all the inclusion criteria.

### Part I—Levels of Physical Activity

The IPAQ is a questionnaire that assesses self-reported physical activity levels; it has been validated worldwide (IPAQ group, 2005) and in different populations (Silsbury et al., 2015; Lopes et al., 2017; Guthold et al., 2018). The IPAQ-SF, a short form of IPAQ, has been recommended for large population-based studies and has been validated for the Portuguese population ranging from 15 to 69 years-old (Campaniço, 2016). Based on 7 items, the IPAQ-SF considers the time and energy expended (metabolic equivalent of task, MET-min⋅week^−1^) in vigorous-intensity activities, moderate-intensity activities and walking, which were done for more than 10 min in the prior 7 days. MET levels of 3.3, 4.0, and 8.0 were attributed for walking, moderate and vigorous intensity activities, respectively. Each participant was assigned to one of three categories: (a) highly active if Vigorous physical activity in 3 or more days a week, accumulating at least MET-min⋅week^−1^ or 7 days or more in which the combination of moderate/vigorous activity or walking cumulate at least 3,000 MET-min⋅week^−1^), (b) moderately active if 3 or more days of vigorous physical activity per week (more than 20 min/week) or more than 4 days with moderate physical activity and/or walking (at least 30 min/day) or 5 or more days with a combination of vigorous/moderate physical activity or walking that cumulate ≥600 MET-min⋅week^−1^) or, (c) low active (when the participant did not any physical activity or it is insufficiency to meet the criteria for highly or moderately active categories) (IPAQ group, 2005). Total MET-min⋅week^−1^ variable was calculated adding individual MET-min⋅week^−1^ for each activity intensity: vigorous, moderate and walking. As proposed by the IPAQ-SF scoring protocol guidelines (IPAQ group, 2005), values of less than 10 min were recoded as “0” and values greater than 960 min of physical activity were truncated to 960 min. Thus, when physical activity variables (i.e., vigorous and moderate physical activity and walking) exceeded 180 min/day, they were truncated to 180 min only. The IPAQ-SF also includes a question about sitting time. This question was: “During the last 7 days, how much time (hours per day and minutes per day) did you spend sitting on a weekday?”, which analyzed separately from the questions included in the score of physical activity. Finally, the levels of physical activity were analyzed according to the recommendations on physical activity and sedentary behavior proposed by the World Health Organization (2020). Bouts of less than 10 min of physical activity were recoded following the IPAQ-SF scoring protocol.

### Part II—Habits and Motivations for Physical Training

To evaluate the habits and motivations of virtual football players, a set of questions (i.e., “What are the main reasons for physical training?”, “Who plans your physical training program?”, “Which influence as physical training on your esports performance?”) were structured based on a previous study (Kari and Karhulahti, 2016). Questions were added to estimate the time dedicated to virtual football game play, the time spent with screen time, and five multiple-choice questions regarding the habits of physical activity. The participants were asked about physical training achievement, the person or professional responsible for physical training, reasons for doing physical training, perception of the effect of doing physical training on esports performance and about the perception of physical training compared to other esports players. In all the questions where none of the possible answers applied, an option “cannot say” was included. The questions were previously translated by an official translator from English to Portuguese, and then externally validated by an English and Portuguese native speaker and by two English-native speakers.

### Data Analysis

Data analysis was done separately for Part 1 (*n* = 433) and Part 2 (*n* = 565). R (R Core Team, 2019) was used to analyze the data collected. Considering the IPAQ-SF guidelines for data processing and normality distribution tests, that proved non-normality, quantitative variables (i.e., age, time, and energy spent with physical activity and time spent in sedentary activities) were described with median and IQR, first and third quartiles (Q1, Q3). Categorical variables were described using absolute (n) and relative (%) frequencies. For comparative analysis, Chi-square was used and a statistical value of α (α = 0.05) was considered.

## Results

The median age of the responders was 22 (Interquartile Range, IQR = 8) years-old, with 98% (*n* = 916) being men. Participants who did not report all the IPAQ-SF questions, nor did answer all applicable questions related to habits of physical training were excluded. This resulted in 433 valid answers for Part I, with 429 (99%) responses from men and 4 from women (1%), and 565 valid answers for Part II, with men responding in 99% (*n* = 557) of the cases.

### Part I—Levels of Physical Activity

**FIGURE 1:**
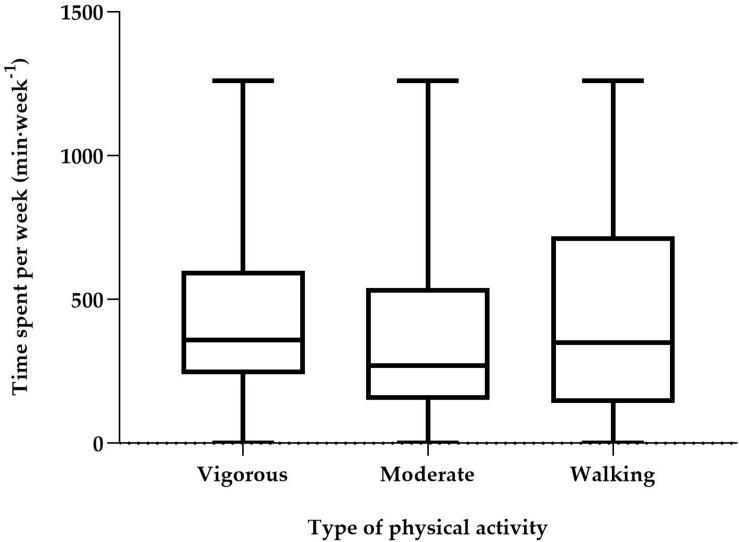
Time spent per week (min⋅week^– 1^) with physical activities, presented by type of physical activity, based on IPAQ-SF. Values are median and interquartile range.

The respondents reported spending a median of 1,080 (Q1, Q3 = 720, 1,680) min/week on physical activities, equivalent to an average of 154 min/day (Figure 1). A median of 320 (210, 480) min was spent in sedentary activities during work or leisure time (e.g., working on the computer, reading, watching television, etc.) each day (Figure 1).

**FIGURE 2:**
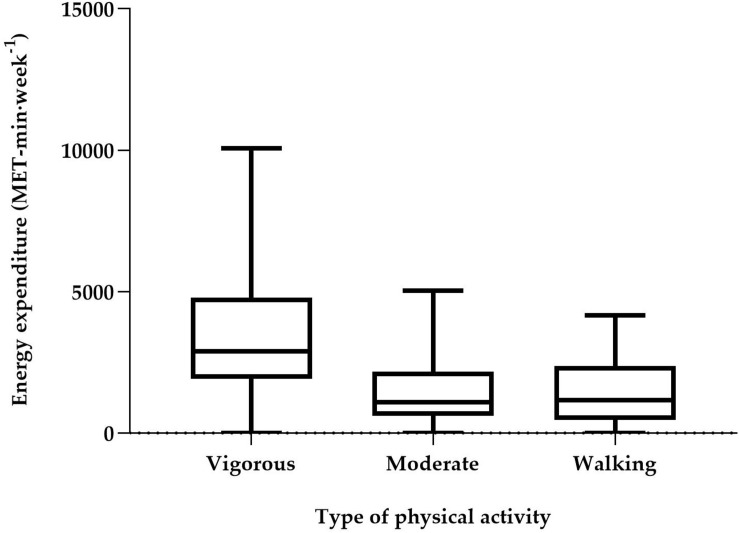
Total energy expenditure (MET-min⋅week^– 1^) in each type of physical activity, based on IPAQ-SF. Values are median and interquartile range.

The total energy expenditure with physical activities was 5,625 (3,675, 8,586). The MET-min⋅week^–1^ participants reported expending 2,880 (1,920, 4,800) MET-min⋅week^−1^ with vigorous activities, 1,080 (600, 2,160) MET-min⋅week^−1^, moderate activities and 1,155 (462, 2,376) MET-min⋅week^−1^ walking (Figure 2).

Following the guidelines for data processing and analysis of the IPAQ-SF, 84.53% (*n* = 366) of the participants reported high levels of physical activity, while 12.93% (*n* = 56) reported moderate levels, and 2.54% low levels of physical activity (*n* = 11). Levels of physical activity differed between age groups, with 90.08% (*n* = 118) of virtual football players younger than 20 years-old responding that high levels of physical activity comparing with 55.00% on the group between 35 and 39 years old (Table 1). Considering the WHO recommendations for physical activity (World Health Organization, 2020), 87.07% (*n* = 377) of the participants met those recommendations, with no significant differences between age groups (*p* > 0.05).

**TABLE 1:** Levels of physical activity stratified by age group.

Age group (years-old)	Level of physical activity [*n* (%)]		
	
	Low	Moderate	High
<20	2 (1.53)	11 (8.40)	118 (90.08)
20–24	4 (3.05)	15 (11.45)	112 (85.50)
25–29	2 (2.20)	15 (16.48)	74 (81.32)
30–34	1 (2.08)	5 (10.42)	42 (87.50)
35–39	2 (10.00)	7 (35.00)	11 (55.00)
≥40	0 (0.00)	3 (25.00)	9 (75.00)

**Levels of physical activity based on IPAQ—short form scoring protocol: Low level—no physical activity or insufficient to meet the other levels; Moderate Level—3 or more days of vigorous physical activity per week (more than 20 min/week) or more than 4 days with moderate physical activity and/or walking (at least 30 min/day) or 5 or more days with a combination of vigorous/moderate physical activity or walking that cumulate 600 or more MET-min⋅week^−1^; High Level—Vigorous physical activity in 3 or more days a week, cumulating at least 1,500 MET-min⋅week^−1^ or 7 days or more in which the combination of moderate/vigorous activity or walking cumulate at least 3,000 MET-min⋅week^−1^. (χ^2^ = 20.588, *p* = 0.024).**

### Part II—Esports Practice and Physical Training

**FIGURE 3:**
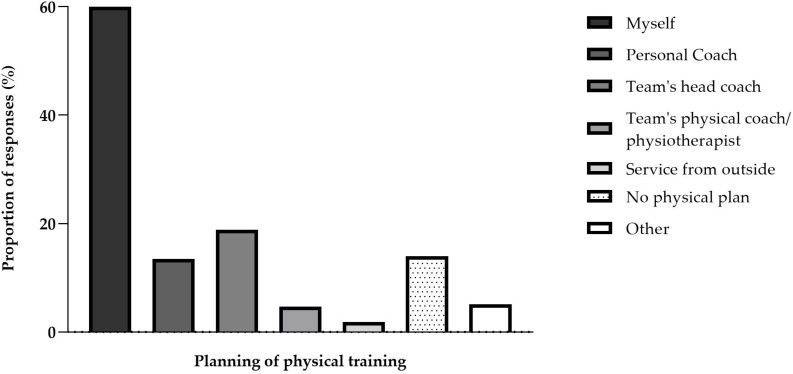
Responses to the question “Who plans your physical training program?”. More than one response was accepted.

The participants reported spending a median of 150 (120, 220) min/day in virtual football practice and 97.70% of the participants responded practicing esports at least for 10 min a day. Interestingly, 76.11% (*n* = 430) of the participants stated to perform regular physical training. Of those, 258 (60.00%) reported planning their physical training themselves, followed by those who had their training planned by the esports team’s coach (*n* = 81; 18.84%), a personal trainer (*n* = 58; 13.49%), the team’s physical coach/physiotherapist (*n* = 20; 4.65%) or an outside service (*n* = 8; 1.86%) (Figure 3).

**FIGURE 4:**
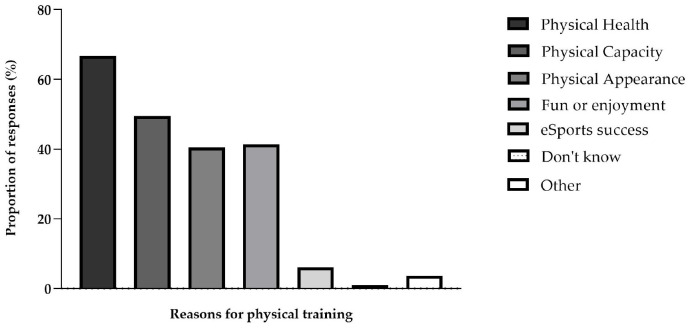
Responses to the question “What is your main reason for doing physical training?”. More than one response was accepted.

Only 26 (6.05%) participants referred doing physical training to be more successful in esports. The most prevalent reason for doing physical training was maintaining or improving overall physical health (*n* = 287; 66.74%). Also, 213 (49.53%) participants reported doing physical activity to improve overall physical capacity. Exercising for fun or enjoyment or improving physical appearance were motivations for 178 (41.40%) and 174 (40.47%) participants, respectively (Figure 4).

Despite a low percentage of respondents stated to partake in physical exercise to improve esports performance, 162 (38.66%) participants reported that doing physical training positively affected their performance. A total of 189 (45.11%) participants considered that physical activity had no effect on their esports performance, while 20 (4.77%) even considered that physical activity had a negative effect.

## Discussion

Esports game play is a contemporary phenomenon wrapped in feverous discussion, with the absence of physicality of esports and its assumed association with increased sedentary behavior being a conflicting topics (Jenny et al., 2016; Pereira et al., 2019). To our knowledge, this is the first investigation on physical activity levels exclusively conducted in virtual football players. The results obtained in this preliminary cross-sectional investigation demonstrated that, on a normal day, the time spent in physical activities was higher than the time spent being sedentary or even practicing esports. Even knowing that the data presented here are only related to virtual football, the results might be a small indicator that the nature of virtual football might not be related to an inactive lifestyle and this preliminary study could open the door to new and more elaborated studies.

### Physical Activity

In the recent guidelines on physical activity and sedentary behavior proposed by the WHO, physical activity is defined as any bodily movement produced by skeletal muscles that requires energy expenditure (World Health Organization, 2020). Being physical active has been associated with lower rates of negative health outcomes and mortality, for both energy expenditure (Gerovasili et al., 2015; Lear et al., 2017; Zhao et al., 2019) and time spent with physical activity (Dyrstad et al., 2014; Stamatakis et al., 2019b). In the current study, 87% of the virtual football players reported meeting the recommendations for time spent with physical activity for adults released by the WHO (World Health Organization, 2020).

When analyzing physical activity patterns, walking and activities with vigorous intensity were the main activities done by the virtual football players. The median of total energy expenditure with physical activities reported was 5,625 MET-min.week^–1^, a value that has been associated with enhanced health-related benefits (Ekelund et al., 2019). The results positively differ from those reported in previous studies that evaluated physical activity levels in adults. In Portugal, Teixeira et al. (Teixeira et al., 2019) they found that 58% of the Portuguese population, was classified as moderately or highly active, while other authors, studying the Portuguese population reported that 70% of participants, aged 18–29 years, reached the recommendation of 30 min/day of physical activity (Clemente et al., 2016). Different studies also noted that men tend to exercise more, during more time and/or with higher intensities than women or older people (Clemente et al., 2016; Bergier et al., 2017; European Commission, 2018; Guthold et al., 2018; Zhao et al., 2019). This could partly explain our results regarding the time spent with physical activity, energy expenditure and levels of physical activity and inactivity, since the current study comprised mostly young men. Further, virtual football players practice a sports-related videogame, an esports genre that has been associated with exercising more, comparing with other videogame genres, such as Role Playing Games (Ballard et al., 2009; Schmidt et al., 2018). The surprisingly high levels of physical activity reported here might be important for understanding health status. Conflicting results regarding levels of physical activity in esports players from different esports genres as an all have been proposed (Kari and Karhulahti, 2016; Trotter et al., 2020). Remarkably, a recent study with 1,722 esports players from different esports genres and skill levels showed that esports players with a higher in-game rank were more physically active when compared to the remaining esports players (Trotter et al., 2020). Likewise, since esports players, including some of the top-level virtual football players, are followed by millions of people worldwide (Cunningham et al., 2018; WePC, 2019), their high physical levels and physically active profile might influence others to follow the same steps.

The reported time spent in sedentary activities was comparable to other studies conducted with the Portuguese adults (i.e., average of 327 min/day) (Teixeira et al., 2019) or athletes (i.e., 500 min/day in runners and footballers) (Exel et al., 2019). Nevertheless, it would be important to discriminate the type of sedentary activities in this population (e.g., reading, screen time, work practice) during esports game play. Interestingly, the WHO now differentiates time spent watching screen-based entertainment (e.g., TV, computer, mobile devices) from active screen-based games where physical activity or movement is required (World Health Organization, 2020).

For instance, sedentary habits such as TV viewing and cell phone use might increase total time spent sitting (Weiler et al., 2015; O’Donoghue et al., 2016; Stamatakis et al., 2019a), which has been associated with increased all-cause mortality (Hallal et al., 2012; U.S. Department of Health and Human Services, 2018).

The time spent with virtual football practice was reported to be 150 min/day. To our knowledge, there is no official data or epidemiological studies on the time spent practicing esports to improve performance. Based mostly on anecdotal and media reports, we believe differences might exist between elite and recreational esports players, and those differences may change with different esports genres. Still, at the time of data collection, as anecdotally reported by the FPF Esports department, the number of Portuguese professional virtual football players was still low and separating professional from recreational players seemed to add no additional value.

### Esports Practice and Physical Training

Sixty percent of the virtual football players reported planning their physical training by themselves. Yet, an exercise professional was chosen in 39% of cases. With the increased professionalization of esports, exercise and sports professionals may need to be prepared for the particularities and special needs of esports players, to help esports players minimizing problems related to their activity, like game-related injuries (Pereira et al., 2019).

In line with other studies on motivation for doing physical activity (Nielsen et al., 2014; European Commission, 2018) maintaining or improving overall physical health was the main reason for physical training. Interestingly, only 6% of the virtual football players reported that improving esports performance was the main motive for doing physical activity, but 39% of the participants agreed that doing physical training positively affects their performance in esports. Kari and Karhulahti (2016) also showed that 9% of elite esports players (from several esports genres) reported the main reason for being active was improving esports performance and 6% believed that integrating physical exercise in their training programmes has a positive effect on esports performance. Actually, recent studies highlighted the association between physical activity and increased esports performance (i.e., accuracy and objective fulfilment, and cognitive aspects of gaming) (de Las Heras et al., 2020; Toth et al., 2020). The fact that the current study included both elite and recreational virtual football players might be a reason to different perceptions to that question.

### Strengths and Limitations

The current preliminary study has some strengths and limitations that we would like to highlight. As previously stated, this is the first study on physical activity habits of virtual football players conducted in Portugal. At the time of the investigation, 5,748 virtual football players were registered in FPF Esports platform, and only 916 players accepted to voluntarily participate in the investigation. Hence, even with a significant number of registered players, the registration process of the FPF Esports platform did not require regular esports participation, meaning that only a small number might be really participating in esports competitions. That initial pool might not have comprised active members or even people who were really engaged with esports. Thus, this was a convenience sample rather than the total population of esports players. Though, we believe those who responded to the questionnaire are the ones that usually use their e-mail to esports-related information (e.g., tournament registration, competition calendar, etc.), hence, not one-time players. Moreover, this was the first scientific study conducted with FPF Esports players. The participants might differ from other participants in traditional sports in the FPF (e.g., soccer players) that are used to participate in surveys and scientific research. At the beginning of the study, limited information was available on how this population would be accepting or leading with questionnaires, or even if electronic questionnaires would be a good fit to their characteristics. Also, the way the survey was conducted (i.e., anonymous) did not allow on having direct follow-up on non-responders and incite the participants to respond. Again, we were not able to have a randomly selected sample which might have increased the introduction of biases (i.e., selection and allocation bias). Though, the players more interested in physical activity might have been the ones more prone to respond to the questionnaire (i.e., volunteer bias) (Delgado-Rodriguez and Llorca, 2004; McPeake et al., 2014; World Health Organization, 2020). Therefore, generalization and external validity might be compromised (Delgado-Rodriguez and Llorca, 2004).

Another arguable problem with this study was the surprisingly high levels of physical activity reported and the proportion of players meeting the WHO’s recommendations for physical activity for adults. To evaluate physical activity levels, we used the Portuguese short version of the IPAQ. Different studies reported an acceptable validity and reproducibility (Helmerhorst et al., 2012; Berman et al., 2015). Even using anonymous on-line questionnaires, the virtual football players could have overreported positive behavior (i.e., physical activity) and underreported negative behavior (i.e., sedentary activity).

Finally, this is a cross-sectional study; it is not possible to establish causal relations or formally test hypotheses. Different reasons, such as a complex competition schedule, existence of different tournaments at the same time, short competition periods of 1–2 days or on-line tournaments determined the use of electronic questionnaires, instead of more robust methods to measure physical activity, such as, accelerometers. Still, for the purpose of a baseline description and the fact that research on esports players is relatively new, the use of questionnaires might be an important approach to start.

In conclusion, virtual football players revealed high levels of physical activity, with 87% meeting the WHO’s recommendations for physical activity. Even though, most of the virtual football players were responsible for their own physical training. Notably, many resorted to an exercise professional for their physical training. Improving overall health, rather than esports performance, was the main motivation for physical activity.

## Data Availability Statement

The raw data supporting the conclusions of this article will be made available by the authors, without undue reservation.

## Ethics Statement

The studies involving human participants were reviewed and approved by the Ethics Committee of the Faculty of Sports, University of Porto (CEFADE/31/2019). The patients/participants provided their written informed consent to participate in this study.

## Author Contributions

AMP, PF, and JB: conceptualization. AMP and AM: data curation. AM: formal analysis and software. AMP and JB: investigation and visualization. AMP, PF, and JB: methodology. AS and JB: project administration and resources. EV and JB: supervision. AMP, PF, and AM: validation. AMP: writing—original draft. AMP, EV, PF, and JB: writing—review and editing. All authors contributed to the article and approved the submitted version.

## Conflict of Interest

The authors declare that the research was conducted in the absence of any commercial or financial relationships that could be construed as a potential conflict of interest.
